# Effects of Various Rice-Based Raw Materials on Enhancement of Volatile Aromatic Compounds in *Monascus* Vinegar

**DOI:** 10.3390/molecules26030687

**Published:** 2021-01-28

**Authors:** Xi Yuan, Xiaoyuan Chen, Muhammad Safiullah Virk, Yinglun Ma, Fusheng Chen

**Affiliations:** 1 Hubei International Scientific and Technological Cooperation Base of Traditional Fermented Foods, Huazhong Agricultural University, Wuhan 430070, China; yuanxiraiden@163.com (X.Y.); safiullahvirk@hotmail.com (M.S.V.); 2 College of Food Science and Technology, Huazhong Agricultural University, Wuhan 430070, China; 3 Nutrition & Health Research Institute, COFCO Corporation, Beijing 102209, China; chenxiaoyuan@cofco.com; 4 Fujian Yongchun Ageing Vinegar Vinegar Industry Co., Ltd., Quanzhou 362000, China; gongyi_2006@163.com

**Keywords:** *Monascus* wine, *Monascus* vinegar, filtrate efficiency, volatile aroma compound

## Abstract

*Monascus* vinegar (MV), during whose brewing process *Monascus* spp. and polished rice (PR) are normally used as the starter and the raw material, respectively, is one of the traditional vinegars in China. In this study, the effects of three raw materials, including PR, unhusked rice (UR), and germinated UR (GR), on MV volatile compounds have been investigated. The results revealed that MV of GR (GMV), and its intermediate *Monascus* wine (GMW), exhibited the highest amount of aroma, not only in the concentrations but also in the varieties of the aromatic compounds mainly contributing to the final fragrance. Especially after three years of aging, the contents of benzaldehyde and furfural in GMV could reach to 13.93% and 0.57%, respectively, both of which can coordinate synergistically on enhancing the aroma. We also found that the filtering efficiency was significantly improved when UR and GR were applied as the raw materials, respectively. Therefore, GR might be more suitable raw materials for MV.

## 1. Introduction

Vinegar is one of the most commonly used acid condiments all over the world. In western countries, vinegars are usually made from fruits (e.g., grape and apple) by the liquid-state fermentation process (LFP), so they are called as fruit vinegars [1]. In eastern countries such as China, Japan, and Korea, vinegars are mainly made from rice, sorghum, corn, wheat, or other starchy cereals, mainly by the solid-state fermentation process (SFP), so they are called as cereal vinegars [2]. In China, Shanxi aged vinegar, Zhenjiang aromatic vinegar, Baoning bran vinegar and Yongchun *Monascus* vinegar (YMV) are known as the four most famous Chinese-type vinegars due to their special local features (e.g., raw materials, local climate and special production process) [3].

In addition to being a condiment, vinegars have both nutritional value and therapeutic effects [4]. It has been proven that vinegars, especially traditional ones, can function as agents of anti-bacteria, anti-infection, anti-oxidation, anti-cancer, blood glucose control, lipid metabolism regulation, weight loss, and so on [5]. The functional compounds in vinegar can be from the raw materials, microorganisms, and the brewing processes [5,6,7]. For example, during YMV production, *Monascus* spp., one of the main microorganisms from the starter, black-skin-red-koji (BSRK), also namely Wuyihongqu in Chinese, can produce many functional metabolites such as *Monascus* pigments, monacolin K, dimerumic acid and γ-aminobutyric acid [8]. Therefore, YMV is also considered a functional food, which can exert hypolipidemic activity and beneficial effects on cardiovascular diseases [9,10].

For cereal vinegars, their production processes mainly include three steps, namely saccharification, alcoholic fermentation and acetic acid fermentation, and the first two steps often happen simultaneously. In the traditional way, all these processes for Shanxi aged vinegar, Zhenjiang aromatic vinegar, and Baoning bran vinegar are in solid-state, while for YMV are in liquid-state [3]. Compared with SFP, LFP has several advantages, such as short fermentation time, high yield, and low cost [11,12]. However, normally the aroma in the LFP vinegar is poorer than that in the SFP vinegar [2,13], so usually aging is an essential process for LFP vinegars and also for some famous SFP vinegars to modify the vinegar flavors by reducing pungent smell and harsh taste of the fresh vinegars [14].

In *Monascus* vinegar (MV) production by LFP, polished rice (PR) is the raw material. An unpublished investigation from YMV industry reveals that the filtration difficulty after alcoholic fermentation is one of the biggest obstacles due to the starch residue in broth, which may be solved or partly solved if unhusked rice (UR) is used to replace PR since, in beer brewing, the barley husk from malt does play an important role in wort filtration [15]. In addition, some studies have showed that rice husk is the source of many aroma active compounds contributing for the aroma of the final products, such as butanal, octanal, and furfural in Chinese Baijiu production [16]. Moreover, germinated unhusked rice (GR) shows impressive health promoting effects [17].

In current study, the effects of three raw materials, including PR, UR, and GR, on MV flavor compounds have been investigated by head space solid-phase microextraction (HS-SPME) coupled with gas chromatography-mass spectrometry (HS-SPME/GC-MS) [18]. The effects of the raw materials on the MW filtering efficiency were also analyzed. It is proven that GR is an appropriate raw material on enhancing aroma of MV while solving the filtration problem.

## 2. Results and Discussion

### 2.1. Effects of Raw Materials on MV Brewing Processes

In present research, the fermentation processes of all tested groups are identical, the only variables are raw materials: PR, UR, and GR. The results showed that all the selected raw materials were successful in the application to brew *Monascus* wines (MWs), the MV intermediates, including PMW, UMW, and GMW, and the final products of MVs, including PMV, UMV, and GMV. All these MWs and MVs presented the unique aroma evaluated by human olfaction.

#### 2.1.1. Saccharification and Alcoholic Fermentation

**Figure 1:**
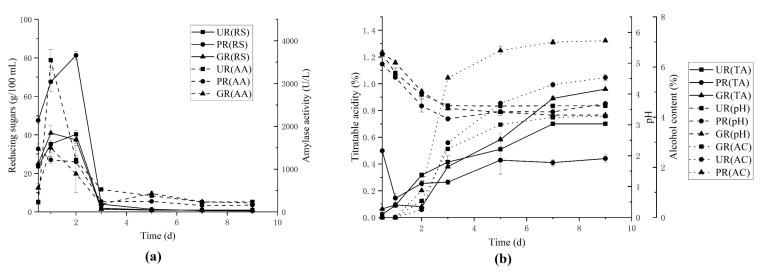
Fermentation traits comparison of three raw materials in brewing MW: (**a**) Comparison of RS contents and AA; (**b**) comparison of AC, TA, and pH value. Abbreviations: PR-polished rice, UR-unhusked rice, GR-germinated rice, RS-reducing sugars, AA-amylase activity, TA-titratable acidity, AC-alcohol content.

During saccharification and alcoholic fermentation (SAF), the reducing sugars (RS) of all three tested groups were increased for the first two days and then decreased (Figure 1a). The UR and PR groups reached the highest concentration of RS after 2 d while GR reached the top after 1 d. The RS was almost depleted after 3 d for all tested groups.

At the beginning of SAF, the microbes mainly including *Monascus* spp., *Aspergillus niger* and yeasts from BSRK starter [19], began to grow and produce amylase, which hydrolyzes starch from the raw material into RS, resulting to its contents in broth increasing rapidly. Hereafter, a part of RS was used for microbial growth, the rest was transferred into alcohols by yeasts, leading to the significant decline of RS at day 3. The content of RS in the UR group was higher than those in the other two groups (Figure 1a). This is probably because of a barrier created by the rice husk and hindered the contact between amylase and starch from raw materials at the initial stage (≤3 d), meanwhile the GR group had the lowest RS content on account of the carbohydrate consumption during rice germination process and leaving less available carbohydrates for conversion [20].

As shown in Figure 1a, the amylase activities of both UR and GR groups were increased during 1st day and reached the highest at 24 h, then decreased immediately. The amylase activity in the GR group was the highest in the start of SAF and decreased with the fermentation course. The possible reasons for this change of amylase activities are as follows: (1) Starters contain microorganisms, such as *A. niger* and *Monascus* spp., which can produce a large amount of amylase; (2) the bacteria such as lactic acid bacteria in SAF can transfer alcohols into acids which make pH value decrease rapidly, leading to amylases unstable [21]. The activity of amylase could also be influenced by the substrates consumption and alcohols production.

At the stage of SAF, the alcoholic contents in broth were slowly accumulated during 0–2 d, rapidly increased during 2–4 d, and gradually increased and stabilized after 4 d, as shown in Figure 1b. And the profile of alcohol content showed an “S” shape. No alcohol could be detected at the beginning of SAF, as the microbial growth, the starches in the raw materials were broken down into RS, then RS was converted to alcohol with the growth of yeasts. Due to the decrease of RS content, the conversion of RS to alcohol gradually decreased. Finally, the alcohol content in the broth was stabilized.

The titratable acidities and pH values of the broth were determined, and the results (Figure 1b) showed a slow raise of titratable acidities and a decrease of pH values. During SAF, *A. niger* and *Monascus* spp. were the predominant microbes at the initial brewing stage [19]. *A. niger* can not only produce a large of amylase to offer RS for other microbes but also produce acid such as citric acid, which can decrease pH value [22]. After RS was produced, other microorganisms especially acid-producing bacteria such as acetic acid bacteria (AAB) and lactic acid bacteria began to grow, and acetic acid and lactic acid were accumulated, resulting in the increase of titratable acidities and decrease of pH values.

#### 2.1.2. Acetic Acid Fermentation

**Figure 2:**
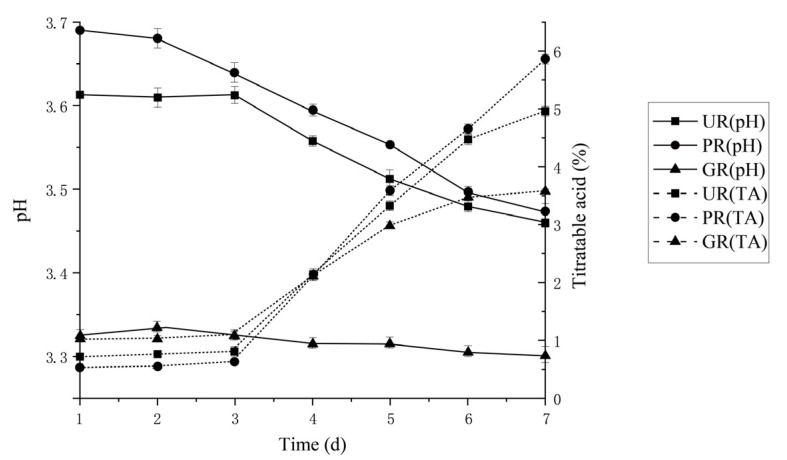
Comparison of pH values and titratable acidities during acetic acid fermentation of *Monascus* vinegars by three raw materials, respectively. Abbreviations: PR-polished rice, UR-unhusked rice, GR-germinated rice, TA-titratable acidity.

Acetic acid fermentation (AAF) is a stage to produce organic acids, mainly acetic acid which is the dominant compound in vinegars [12]. In this stage, the initial titratable acidity and initial pH were different. Titratable acidity was changed a little in the first three days, and then rapidly rose and reached the highest point at 7 d while pH value barely changed (Figure 2). In AAF, AAB is responsible for the transformation of ethanol to acetic acid [23], so after inoculation of *Acetobacter pasteurianus* As1.41, it started to consume of RS, and grew slowly during the first three days and did not convert a large amount of ethanol into acetic acid. After 3 d, the As1.41 strain began to convert a greater amount of ethanol into acetic acid, leading to a rapid rise of the titratable acidity in the broth, which reached its maximum at day 7, while pH value did not change significantly, since acetic acid is organic weak acid, which had no significant influence on the concentration of free hydrogen ion [H^+^] in the broth.

### 2.2. Improvement of Filtrate Efficiency

**Figure 3:**
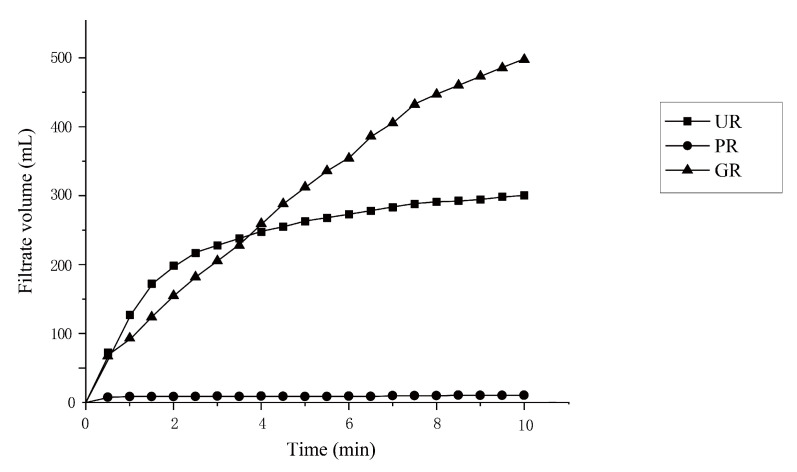
Filtering velocities of *Monascus* wines produced by three raw materials. Abbreviations: PR-polished rice, UR-unhusked rice, GR-germinated rice.

After SAF, the filtration velocity of the broth was analyzed and compared. From the results in Figure 3, it was obvious that the broth of PR group had an inefficient filtering capacity mainly caused by the sticky undecomposed starch, while the UR group had a very fast initial filtration velocity, slowed down gradually, and stabilized eventually, and the overall filtering velocity of the GR group was fast. All these results showed that the filtering layer of rich husk in UR and GR groups can improve the filtration velocity and solve filtering problem perfectly. Similarly, in beer brewing, the husk of barley can also play an important role in malt lautering and filtration [15].

### 2.3. Aroma Compounds Analysis by HS-SPME/GC-MS

#### 2.3.1. Aroma Compounds Analysis and Comparison in *Monascus* Wines

HS-SPME/GC-MS was used to determine the volatile aroma compounds in the intermediate products, MWs of MVs. According to the volatile aroma compounds’ results of PMW, UMW and GMW (Supplementary Materials, Table S1), after 9 d of SAF, 28, 27, and 28 volatile compounds were detected in PMW, UMW, and GMW, respectively. Depending on the subjective sense of smell, the aroma of the three obtained MWs ranked from good to bad was GMW, PMW, UMW. More precisely, UMW even had an unpleasant sour smell.

**Figure 4:**
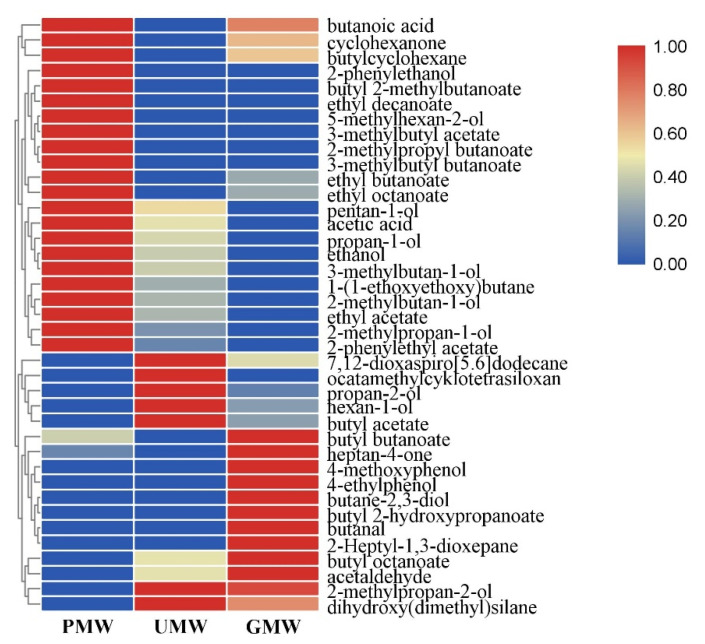
Heatmap of volatile compounds in three obtained MWs. PMW, *Monascus* wine using polished rice as a raw material; UMW, *Monascus* wine using unhusked rice as a raw material; GMW, *Monascus* wine using germinated rice as a raw material.

A hierarchical clustering of aroma compounds was performed to illustrate volatile compounds in different MWs (Figure 4). Compared to traditional PMW, 17 volatile compounds were new detected in UMW and GMW. The results revealed that the volatile aroma compounds are very complex, mainly including alcohols, aldehydes, phenols, acids, and esters. The key aroma compounds of MWs were mainly volatile alcohols and esters [24]. In UMW, the total alcohol contents more than 90% while the total esters contents were only 5%. In PMW and GMW, the total alcohol contents were not as high as UMW, but the total esters contents reached to 13.25% and 16.55%, respectively. The volatile compound profile in UMW group was more inferior than those in the other groups both in number and species, and even the unique volatile odorless compound octamethylcyklotetrasiloxan [25]. The contents of total esters, especially butyl butyrate which is one of the short-chain esters known as a flavor and fragrance compounds has strong flower and fruit aroma [26], was higher in GMW than those in PMW. Aldehydes such as acetaldehyde and butanal were unique in GMW. And the aldehydes can provide almond and sweet flavor [27] and coordinate the release of aroma.

At the initial stage of SAF, the contents of alcohol were high, while the contents of esters were very low. With the elongation of fermentation time, alcohols and aldehydes in the wine were oxidized, resulting in the gradual increase in organic acids. In this study, the fermentation duration was comparatively short, only nine days, and the organic acid contents were low. Therefore, fewer corresponding esterification reactions occurred in the broth, resulting in the lower contents of total esters in the broth. Normally, aldehydes in rice wine are mainly derived from the oxidation of alcohol during the aging process [28], but the three obtained MWs only have undergone the nine days of alcoholic fermentation process, without aging process. Thus, compared to PMW and UMW, the detected aldehydes and phenols in GMW may probably related to substances produced during the germination process of rice.

#### 2.3.2. Aroma Compounds Analysis and Comparison of *Monascus* Vinegars

After acetic acid fermentation, according to the volatile aroma compounds’ results of *Monascus* vinegars (MVs) via HS-SPME/GC-MS (Supplementary Materials, Table S2), 40, 35, and 37 volatile substances were found in PMV, UMV, and GMV, respectively.

**Figure 5:**
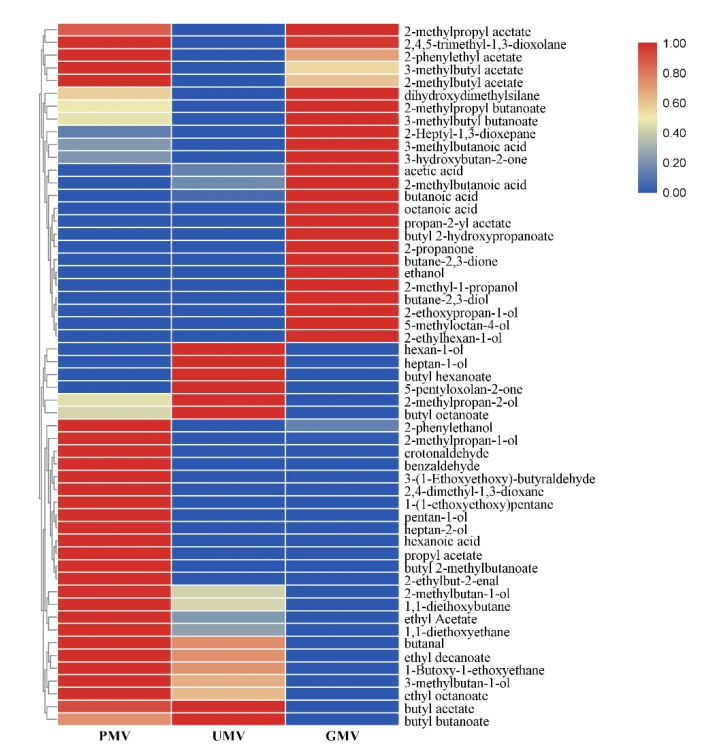
Heatmap of volatile compounds in three obtained MVs. Abbreviations: PMV-*Monascus* vinegar using polished rice as raw material, UMV-*Monascus* vinegar using unhusked rice as raw material, GMV-*Monascus* vinegar using germinated rice as raw material.

A hierarchical clustering of aroma compounds was performed to illustrate volatiles of compounds of different MVs (Figure 5). Compared to traditional PMV, 26 volatile compounds were new detected in UMV and GMV. The results revealed that the esters formed by reaction between organic acids and alcohols were the main aroma components and can enhance the flavor of the vinegar [29]. At the end of acetic acid fermentation stage, the main volatile aromatic substance of PMV and UMV was 1-butanol, while the main volatile aromatic substance of GMV was butanoic acid formed after oxidation of 1-butanol. Although both UMW and GMW contained high content of 1-butanol in their volatile aroma components (Figure 5), only in GMV 1-butanol was completely oxidized to the corresponding acid. After acetic acid fermentation, the esters content was the highest in the traditional PMV, followed by UMV, and the lowest in GMV, may be due to the large amount of organic acid such as acetic acid produced during acetic acid fermentation. Esterification reactions occurred between acetic acid and ethanol in the broth and a large amount of ethyl acetate was produced. These results can match the results of Section 2.1.1 which showed that alcohol content in PMW was the highest, followed by the UMW, and GMW was the lowest. Although the content of total ester substances in GMV was low, some other esters that were specific and had a high concentration were detected, such as propan-2-yl acetate, butyl 2-hydroxypropanoate, 3-methylbutyl butanoate, 2-phenylethyl acetate, and the relatively high content of aldehyde and ketone compounds, such as 3-hydroxybutan-2-one, 2-propanone and butane-2,3-dione, as well as other compounds, such as 2-methoxyphenol, making the overall aroma of GMV as rich as that of traditional PMV.

#### 2.3.3. Effects of Three-Year Aging on Aroma Compounds of PMV and GMV

According to the results given in Section 2.3.1 and Section 2.3.2, UR is not a suitable choice for either MW or MV brewing because of the bad smell and lack of aroma compounds contributing for the fragrance. Therefore, GR and PR were finally used as raw materials in this experiment to compare the changes of volatile aroma compounds in PMV and GMV after three years of aging. The results (Supplementary Materials, Table S3) showed that 26 volatile substances were detected in the three-year aged RMV, including five alcohols, seven esters, five acids, six aldehydes and ketones, and three other substances. A total of 60 volatile substances, including six alcohols, 15 esters, eight acids, 22 aldehydes and ketones, and nine other substances, were detected in three-year aged GMV.

**Figure 6:**
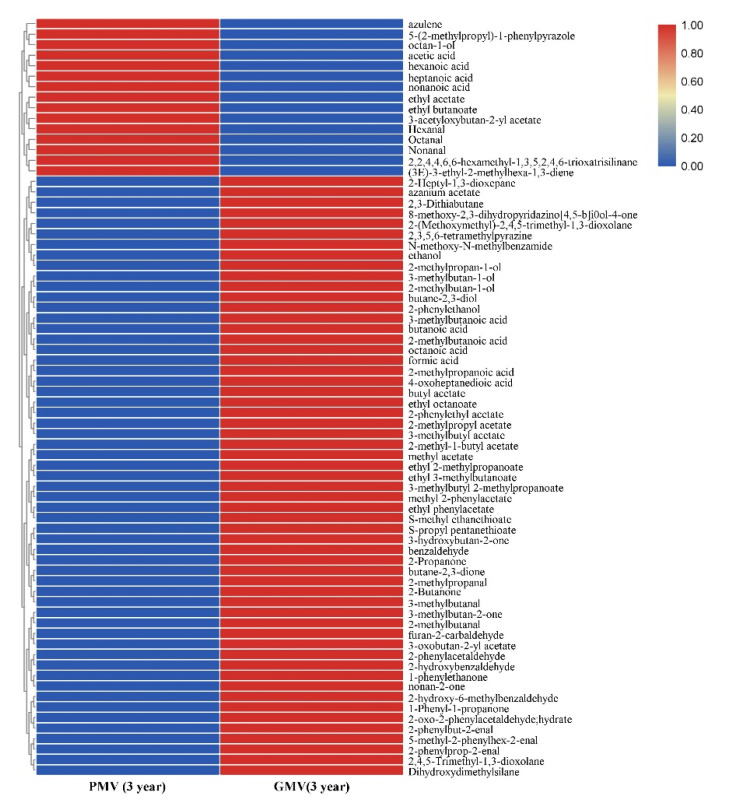
Heatmap of volatile compounds of two three-year aging MVs. Abbreviations: PMV-*Monascus* vinegar using polished rice as raw material, GMV-*Monascus* vinegar using sprouted rice in the husk as raw material.

A hierarchical clustering of aroma compounds was performed to illustrate volatile compounds of two three-year MVs (Figure 6).

The types of volatile substances in PMV after three years of aging were significantly reduced compared with that of before aging. In which acetic acid accounted for 64.37% of the total aromatic components, followed by ethyl acetate 20.59% and a small number of other esters and aldehydes and ketones. The aroma became simpler than that before aging. However, after three years of aging, GMV has undergone tremendous changes in volatile aromatic compounds and its aromatic components were very rich. The contents of esters were higher than that of PMV, and the most significant change was the increase of aldehydes and ketones, among which the contents of benzaldehyde reached 13.93%, while the contents of benzaldehyde in PMV were only 1.97%. Furfural is also a special product in GMV whose contents reached 0.574%. Studies have shown that benzaldehyde mainly formed by the action of microorganisms on aromatic amino acids or the oxidation of benzyl alcohol [27] can provide the fermentation broth a distinctive almond flavor. Furfural can provide almond and burnt sugar flavor. Furfural was the main degradation product of carbohydrates and was usually associated with nonenzymatic browning reactions, namely, the Maillard reaction (MR), sugar degradation and caramelization in acidic media [30]. Furfural can also contribute to the stable pigments in broth which may affect the color of the vinegar. It has been revealed that benzaldehyde can coordinate with furfural synergistically, reducing the overall olfactory threshold value efficiently [28].

From what has been discussed above, changing raw materials can change volatiles compounds of MWs and MVs significantly, even if the tested raw materials are all rice-based. However, for fermented food, not only the materials but also the microorganisms participated in the fermentation can affect the flavor. In bread-making process, the impact of tested yeast even had a greater impact than raw materials [31]. Therefore, based on the traditional brewing process, finding the appropriate microbial resources participated in MV fermentation will be our future work on improving MV flavor.

#### 2.3.4. Principal Component Analysis (PCA) of Volatile Compounds

**Figure 7:**
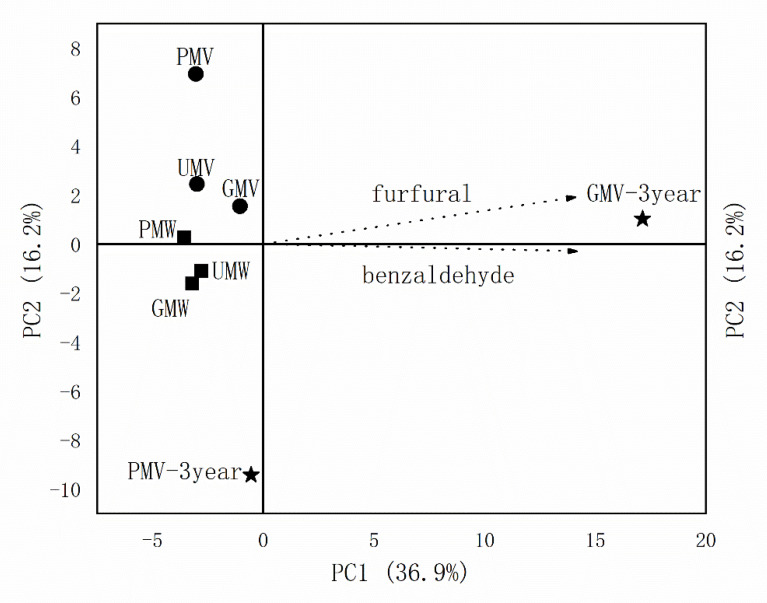
Principal component analysis (PCA) of the obtained MWs and MVs. Abbreviations: ■ MW: PMW-*Monascus* wine using polished rice as raw material, UMW-*Monascus* wine using unhusked rice as raw material, GMW-*Monascus* wine using germinated rice as raw material. ● MV: PMV-*Monascus* vinegar using polished rice as raw material, UMV-*Monascus* vinegar using unhusked rice as raw material, GMV-*Monascus* vinegar using germinated rice as raw material. ★ MV-3 year.

Principal component analysis (PCA) was conducted to distinguish all the obtained MWs and MVs. From the PCA analysis in Figure 7, the accumulated contribution rate of the first two principal components PC1 and PC2 were 36.9% and 16.2%, respectively. Both three-year MVs were separated from others, which illustrated that three-year ageing had significantly changed the volatile compounds of MV. The GMV-3 year exhibited high scores on positive PC1, where the loadings of above-mentioned aroma compounds furfural and benzaldehyde were high.

## 3. Materials and Methods

### 3.1. Raw Material Preparation

Unhusked rice (UR) was bought from Hubei province in China. Polished rice (PR) was achieved from rice by hulling. UR was soaked in the water at 7 °C for 6 h, then transferred evenly on the gauze and cultivated at 20 °C for 4 d, finally the germinated rice (GR) was dried at 40 °C in the oven until the weight was constant. Starters (Black-skin-red-koji, BSRK) were provided by Taoxi Yongchun *Monascus* Vinegar Company (Quanzhou, China). *Acetobacter pasteurianus* As1.41 is preserved in our lab. LB medium (tryptone 10 g, yeast extract 5 g, NaCl 10 g, and 1000 mL distilled water) and GYC medium (glucose 60 g, yeast extract 10 g, CaCO_3_ 30 g, agar 15 g, and 1000 mL distilled water) were prepared for As1.41 cultivation [32].

### 3.2. Fermentation Process

**Figure 8:**
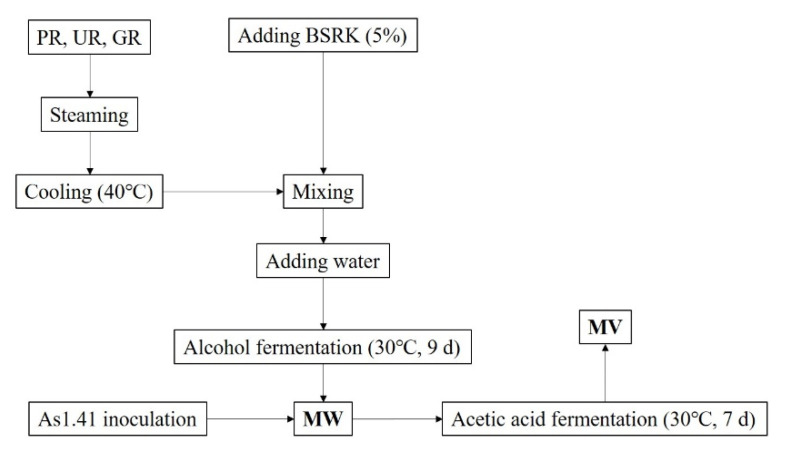
Schematic brewing process of *Monascus* vinegar. Abbreviations: PR-polished rice, UR-unhusked rice, GR-germinated rice, BSRK-black-skin-red-koji, MW-*Monascus* wine, MV-*Monascus* vinegar.

The *Monascus* wine (alcoholic fermentation) and *Monascus* vinegar (acetic acid fermentation) were brewed as the traditional method [6] with some modifications using PR, UR, and GR as main substrates (Figure 8).

For each tested sample, 500 g of raw materials were weighed, washed, soaked, and steamed at 100 °C for 20 min. After the temperature was cooled under 40 °C, 5% starters (BSRK) were added and mixed with the steamed raw materials. Then the substrates were transferred to the jars. Added water to the jar until the total ratio of the steamed material to water was 1:4. The jars were placed in a 30 °C incubator for nine days alcoholic fermentation [3].

After alcoholic fermentation, the broth was filtered and inoculated with 5% *Acetobacter pasteurianus* As1.41 activated broth, which was prepared 3 d earlier using LB medium and GYC medium as culture medium. Afterwards, the jars were placed at 30 for seven days of acetic acid fermentation.

The fermentation traits were compared accordingly by amylase activities, reducing sugars, pH values, titratable acidity, and alcohol contents, which determination methods are as follows.

#### 3.2.1. Reducing Sugars Determination

Reducing sugars was estimated by DNS (3,5-dinitrosalicylic acid) method using glucose as standard [33].

#### 3.2.2. Amylase Activities Determination

Amylase activities were analyzed based on that outlined in Section 3.2.1, using DNS to reflect the variation of reducing sugar which may represent the amylase activities [34].

##### 3.2.3. pH Determination

pH variations during alcoholic and acetic acid fermentation were detected by Sartorius PB-10 pH meter (Sartorius, Göttingen, Germany) [35].

##### 3.2.4. Alcohol Determination

Alcohol contents were detected by the rapid oxidative method using potassium dichromate as an indicator [36]. After the alcohol samples were reacted with potassium dichromate and sulfuric acid, the absorbance values were measured at the wavelength of 600 nm, and the standard curve was drawn. Alcohol contents of samples were calculated according to the standard curve.

##### 3.2.5. Titratable Acidity Determination

The titratable acid was detected by Chinese national standard method (GB/T 5009.41, 2003) counted by acetic acid. Two to three drops of phenolphthalein were added into 50 mL fermentation broth as an indicator then titrated with 0.1 M NaOH solution until light pink color. The titratable acid measured by acetic acid was calculated according to the consumed volume of NaOH [37].

### 3.3. Filtering Efficiency Comparison

*Monascus* wines achieved in Section 3.2 were poured into a container whose bottom contained gauze. For each sample, the filtrate was collected by measuring cylinder and the filtrate volume was measured every 30 s. The gathering speed of the filtrate, which reflects filtering efficiency, was calculated by filtrate volume variation per unit time.

### 3.4. Aroma Components Analysis by Head Space Solid-Phase Microextraction-Gas Chromatography-Mass Spectrometry(HS-SPME/GC-MS)

Aroma components in the samples were extracted by HS-SPME (Supelco, Bellefonte PA, USA) and determined by GC-MS (Shimadzu TQ8040, Shimadzu Corporation, Kyoto, Japan). 5 mL sample was placed in 20 mL head space bottle, 1 g NaCl and magnetic agitator were added and the cap was tightly screwed. The solution was agitated and equilibrated at 40 °C for 15 min, then the fiber was then inserted into the vial septum and exposed to the head space for 40 min at 40 °C. A DB-wax packed with polyethylene glycol capillary column (30 m × 0.25 mm, and 0.25 μm film thickness, Agilent, J&W Scientific, Folsom, CA, USA) was used. The carrier gas was highly pure helium with flow rate of 1 mL/min and separation ratio was 5:1. The injection temperature was 200 °C. The initial temperature of the program was 40 °C, and the temperature was kept for 3 min. The temperature was increased to 120 °C at a rate of 5 °C/min, then increased to 200 °C at a rate of 10 °C/min and the temperature was kept for 5 min. MS conditions: EI ionization source, energy 70 eV, scanning range 30–500 *m*/*z* [38]. Peak area normalization method was performed to reflect aroma compounds variation [39].

### 3.5. Statistical Analysis

The data of all the samples were analyzed by SPSS 18.0 (SPSS Inc., Chicago, IL, USA) and expressed as the mean ± SD. Heatmap was drawn by TBtools [40]. PCA was analyzed and visualized by Origin 2019b (OriginLab Corporation, Northampton, MA, USA).

## 4. Conclusions

This research demonstrated that changing the raw materials may be an alternative way to improve aroma of MW and MV according to the volatile compound analysis by HS-SPME/GC-MS. With the same starters, brewing process, and culture conditions, the MWs and MVs from three selected materials possess significant differences on fragrance not only by human smell but also by HS-SPME/GC-MS. UR may not be an ideal material because of the unpleasant smell and fewer aroma compounds. However, using GR instead of PR, which is a common raw material for MV, can give MV distinct and pleasant smell, owing to the contribution of richer aldehyde and ketones, especially benzaldehyde and furfural, which have been reported to coordinate synergistically on improving fragrance. In addition, the rice husk of GR can overcome the filtering problem which is one of the biggest obstacles to industrial production of MV at present. In summary, this research offered the theoretical basis for MV aroma improving and industrial production solution of the filtrate difficulty, and provided a reference for the further study on the mechanisms about MV aroma compounds variation when changing raw materials. Furthermore, the rice husk contains ferulic acid [41], which is an important functional component in Chinses cereal vinegars [5,42], resulting that GMV can also be developed a potential functional product. Also, the present work only demonstrated the volatile compounds analysis at the given time points. As a direct-injection mass spectrometric (DIMS) technology, proton transfer reaction (PTR), combined with a time-of-flight (ToF) mass spectrometer (MS) may be a potential method to study the dynamic variation of volatile compounds during the whole fermentation of MW and MV (on-line bioprocess monitoring), due to its rapid determination, high sensitivity, and accuracy [43].

## Data Availability

The data presented in this study are available on request from the corresponding author.

## Supplementary Materials

The following are available online, Table S1: Volatile aroma compounds in obtained wines, Table S2: Volatile aroma compounds in obtained vinegars, Table S3: Volatile aroma compounds of three-year PMV and GMV.

## Sample Availability

Not available.
