# Supplementary material for: Effects of Various Rice-Based Raw Materials on Enhancement of Volatile Aromatic Compounds in Monascus Vinegar
Source: Molecules. 2021 Jan 28;26(3):687. doi: 10.3390/molecules26030687 (PMC7866154; doi:10.3390/molecules26030687)
Supplement: Supplementary file 1 [file molecules-26-00687-s001.pdf]

Supplementary Materials

Table S1. Volatile aroma compounds in obtained wines

Number	Compounds	Cas	Relative amount (%)		
			PMW	UMW	GMW
	**Alcohols**				
1	ethanol	64-17-5	33.491	21.403	13.375
2	propan-2-ol	67-63-0	4.737	7.551	5.124
3	propan-1-ol	71-23-8	0.074	0.032	Nd
4	2-methylpropan-1-ol	78-83-1	0.947	0.292	0.127
5	2-methylpropan-2-ol **(1-butanol)**	71-36-3	34.362	58.092	56.430
6	3-methylbutan-1-ol	123-51-3	3.172	1.686	0.683
7	2-methylbutan-1-ol	137-32-6	1.173	0.527	0.221
8	pentan-1-ol	71-41-0	0.055	0.030	Nd
9	2-phenylethanol	60-12-8	1.280	0.568	0.566
10	hexan-1-ol	111-27-3	Nd	0.150	0.035
11	5-methylhexan-2-ol	627-59-8	0.150	Nd	Nd
12	butane-2,3-diol	513-85-9	Nd	Nd	0.032
	Total		79.439	90.329	76.594
	**Acids**				
1	acetic acid	64-19-7	0.112	0.084	0.059
2	butanoic acid	107-92-6	0.310	0.166	0.278
	Total		0.422	0.250	0.337
	**Esters**				
1	ethyl acetate	141-78-6	0.222	0.121	0.073
2	ethyl butanoate	105-54-4	4.683	1.159	2.140
3	butyl acetate	123-86-4	0.975	1.827	1.184
4	3-methylbutyl acetate	123-92-2	0.100	Nd	Nd
5	2-methylpropyl butanoate	539-90-2	0.209	Nd	Nd
6	butyl butanoate	109-21-7	6.298	1.745	12.878
7	3-methylbutyl butanoate	106-27-4	0.131	Nd	Nd
8	butyl 2-methylbutanoate	15706-73-7	0.101	Nd	Nd
9	ethyl octanoate	106-32-1	0.280	0.061	0.124
10	2-phenylethyl acetate	103-45-7	0.123	0.019	Nd
11	ethyl decanoate	110-38-3	0.127	Nd	Nd
12	butyl octanoate	589-75-3	Nd	0.043	0.090
13	butyl 2-hydroxypropanoate	138-22-7	Nd	Nd	0.061
	Total		13.248	4.975	16.551
	**Aldehydes and ketones**				
1	heptan-4-one	123-19-3	0.198	0.062	0.911
2	cyclohexanone	108-94-1	6.218	4.114	5.443

3	acetaldehyde	75-07-0	Nd	0.056	0.119
4	butanal	123-72-8	Nd	Nd	0.176
	Total		6.416	4.232	6.650
	**Others**				
1	1-(1-ethoxyethoxy)butane	57006-87-8	0.246	0.073	Nd
2	butylcyclohexane	1678-93-9	0.144	0.048	0.105
3	7,12-dioxaspiro[5.6]dodecane	181-28-2	0.086	0.568	0.303
4	2,2,4,4,6,6,8,8-octamethyl-1,3,5,7,2,4,6,8-tetraoxatetrasilocane **(ocatamethylcyklotetrasiloxan)**	556-67-2	Nd	0.030	Nd
5	dihydroxy(dimethyl)silane	1066-42-8	Nd	0.063	0.047
6	2-Heptyl-1,3-dioxepane	61732-92-1	Nd	Nd	0.081
7	4-methoxyphenol	150-76-5	Nd	Nd	0.059
8	4-ethylphenol	123-07-9	Nd	Nd	0.096
	Total		0.476	0.782	0.689

Table S2. Volatile aroma compounds in obtained vinegars

Number	Compound	Cas	Relative amount (%)		
			PMV	UMV	GMV
	**Alcohols**				
1	pentan-1-ol	71-41-0	0.037	Nd	Nd
2	ethanol	64-17-5	Nd	Nd	0.067
3	2-methyl-1-propanol	67-63-0	Nd	Nd	3.132
4	2-methylpropan-1-ol	78-83-1	4.089	0.607	0.645
5	2-methylpropan-2-ol **(1-butanol)**	71-36-3	23.303	50.214	Nd
6	3-methylbutan-1-ol	123-51-3	2.470	2.004	1.076
7	2-methylbutan-1-ol	137-32-6	0.992	0.656	0.413
8	heptan-2-ol	543-49-7	0.078	Nd	Nd
9	2-phenylethanol	60-12-8	2.492	1.445	1.594
10	hexan-1-ol	111-27-3	Nd	0.254	Nd
11	heptan-1-ol	111-70-6	Nd	0.021	Nd
12	butane-2,3-diol	513-85-9	Nd	Nd	0.591
13	2-ethoxypropan-1-ol	19089-47-5	Nd	Nd	1.305
14	5-methyloctan-4-ol	59734-23-5	Nd	Nd	0.153
15	2-ethylhexan-1-ol	104-76-7	Nd	Nd	0.191
	Total		33.460	55.201	9.168
	**Acids**				
1	acetic acid	64-19-7	0.202	0.796	4.248
2	hexanoic acid	142-62-1	5.455	Nd	Nd
3	3-methylbutanoic acid	503-74-2	0.097	0.049	0.265
4	butanoic acid	107-92-6	Nd	3.880	54.679

5	2-methylbutanoic acid	116-53-0	Nd	0.031	0.179
6	octanoic acid	124-07-2	Nd	Nd	0.136
	Total		5.754	4.756	59.508
	**Esters**				
1	ethyl Acetate	141-78-6	25.589	11.297	7.256
2	propyl acetate	109-60-4	0.080	Nd	Nd
3	2-methylpropyl acetate	110-19-0	0.553	0.107	0.618
4	butyl acetate	123-86-4	20.321	21.362	7.750
5	3-methylbutyl acetate	123-92-2	1.301	0.549	0.961
6	2-methylbutyl acetate	624-41-9	0.443	0.197	0.347
7	2-methylpropyl butanoate	539-90-2	0.093	Nd	0.188
8	butyl butanoate	109-21-7	1.560	1.983	0.348
9	3-methylbutyl butanoate	106-27-4	0.072	Nd	0.151
10	butyl 2-methylbutanoate	15706-73-7	0.053	Nd	Nd
11	ethyl octanoate	106-32-1	0.161	0.101	Nd
12	2-phenylethyl acetate	103-45-7	0.661	0.222	0.528
13	butyl octanoate	589-75-3	0.037	0.086	Nd
14	ethyl decanoate	110-38-3	0.050	0.037	Nd
15	butyl hexanoate	626-82-4	Nd	0.042	Nd
16	propan-2-yl acetate	108-21-4	Nd	Nd	0.823
17	butyl 2-hydroxypropanoate	138-22-7	Nd	Nd	0.317
	Total		50.972	35.984	19.287
	**Aldehydes and ketones**				
1	3-hydroxybutan-2-one	513-86-0	1.165	0.241	4.607
2	2-ethylbut-2-enal	19780-25-7	0.141	Nd	Nd
3	1,1-diethoxybutane	3658-95-5	0.098	0.042	Nd
4	butanal	123-72-8	0.469	0.351	Nd
5	crotonaldehyde	123-73-9	0.279	Nd	Nd
6	benzaldehyde	100-52-7	0.331	Nd	Nd
7	2-propanone	67-64-1	Nd	Nd	2.185
8	5-pentyloxolan-2-one	104-61-0	Nd	0.090	Nd
9	butane-2,3-dione	431-03-8	Nd	Nd	0.923
	Total		2.482	0.724	7.715
	**Others**				
1	2,4,5-trimethyl-1,3-dioxolane	3299-32-9	2.011	0.119	1.963
2	1,1-diethoxyethane	105-57-7	0.922	0.236	Nd
3	3-(1-Ethoxyethoxy)-butyraldehyde	-	0.218	Nd	Nd
4	2,4-dimethyl-1,3-dioxane	766-20-1	1.069	Nd	Nd
5	dihydroxydimethylsilane	1066-42-8	0.085	0.064	0.101
6	1-Butoxy-1-ethoxyethane	57006-87-8	2.235	1.637	Nd
8	2-Heptyl-1,3-dioxepane	4469-24-3	0.161	Nd	1.199

9	1-(1-ethoxyethoxy)pentane	13442-89-2	0.309	Nd	Nd
10	1-(1-butoxyethoxy)butane	871-22-7	0.324	0.910	Nd
11	1-ethenoxybutane	111-34-2	Nd	0.050	Nd
12	1-(1-ethoxyethoxy)propane	20680-10-8	Nd	0.179	Nd
13	5-methyl-4,6-dioxadecane	-	Nd	0.042	Nd
14	4-ethylphenol	123-07-9	Nd	0.098	0.267
15	1,1-hexylenedioxybutane	-	Nd	Nd	0.238
16	Phenol	108-95-2	0.037	Nd	0.157
17	2-methylpropanoyl 2-methylpropanoate	97-72-3	Nd	Nd	0.169
18	2-methoxyphenol **(o-Guaiacol)**	90-05-1	Nd	Nd	0.229
	Total		7.369	3.335	4.322

Table S3. Volatile aroma compounds of 3-year PMV and GMV

Number	Compound	Cas	Relative amount(%)	
			PMV (3 year)	GMV(3 year)
	**Alcohols**			
1	ethanol	64-17-5	Nd	1.007
2	2-methylpropan-1-ol	78-83-1	Nd	14.689
3	3-methylbutan-1-ol	123-51-3	0.355	0.958
4	2-methylbutan-1-ol	137-32-6	0.392	0.706
5	butane-2,3-diol	513-85-9	Nd	0.655
6	5-(2-methylpropyl)-1-phenylpyrazole	3191-86-4	0.090	Nd
7	octan-1-ol	111-87-5	0.165	Nd
8	2-phenylethanol	60-12-8	1.241	2.729
	Total		2.243	20.744
	**Acids**			
1	acetic acid	64-19-7	64.371	8.693
2	hexanoic acid	142-62-1	0.306	Nd
3	3-methylbutanoic acid	503-74-2	Nd	1.062
4	butanoic acid	107-92-6	Nd	0.146
5	2-methylbutanoic acid	116-53-0	Nd	0.433
6	octanoic acid	124-07-2	0.403	0.421
7	heptanoic acid	111-14-8	0.186	Nd
8	nonanoic acid	112-05-0	0.152	Nd
9	formic acid	64-18-6	Nd	0.028
10	2-methylpropanoic acid	79-31-2	Nd	0.200
11	4-oxoheptanedioic acid	502-50-1	Nd	0.212
	Total		65.419	11.194
	**Esters**			
1	ethyl acetate	141-78-6	20.595	12.941
2	butyl acetate	123-86-4	Nd	0.195

3	ethyl octanoate	106-32-1	Nd	0.125
4	2-phenylethyl acetate	103-45-7	3.784	5.444
5	2-methylpropyl acetate	110-19-0	0.235	1.345
6	ethyl butanoate	105-54-4	0.287	Nd
7	3-methylbutyl acetate	123-92-2	1.485	4.540
8	2-methyl-1-butyl acetate	624-41-9	0.325	2.301
9	methyl acetate	79-20-9	Nd	0.104
10	ethyl 2-methylpropanoate	97-62-1	Nd	0.217
11	ethyl 3-methylbutanoate	108-64-5	Nd	0.275
12	3-methylbutyl 2-methylpropanoate	2050-01-3	Nd	0.223
13	methyl 2-phenylacetate	101-41-7	Nd	0.058
14	ethyl phenylacetate	101-97-3	Nd	0.152
15	3-acetyloxybutan-2-yl acetate	1114-92-7	0.203	Nd
16	S-methyl ethanethioate	1534-08-3	Nd	0.173
17	S-propyl pentanethioate	2432-76-0	Nd	0.122
	Total		26.914	28.215
	**Aldehydes and ketones**			
1	3-hydroxybutan-2-one	513-86-0	1.336	3.241
2	benzaldehyde	100-52-7	1.973	13.929
3	2-Propanone	67-64-1	Nd	0.250
4	butane-2,3-dione	431-03-8	1.455	2.294
5	Hexanal	66-25-1	0.069	Nd
6	Octanal	124-13-0	0.149	Nd
7	Nonanal	124-19-6	0.143	0.119
8	2-methylpropanal	78-84-2	Nd	0.177
9	2-Butanone	78-93-3	Nd	1.352
10	3-methylbutanal	590-86-3	Nd	1.373
11	3-methylbutan-2-one	563-80-4	Nd	0.245
12	2-methylbutanal	96-17-3	Nd	0.453
13	furan-2-carbaldehyde **(furfural)**	98-01-1	Nd	0.574
14	3-oxobutan-2-yl acetate	4906-24-5	Nd	0.341
15	2-phenylacetaldehyde	122-78-1	Nd	0.216
16	2-hydroxybenzaldehyde	90-02-8	Nd	0.286
17	1-phenylethanone	98-86-2	Nd	0.269
18	nonan-2-one	821-55-6	Nd	1.132
19	2-hydroxy-6-methylbenzaldehyde	18362-36-2	Nd	0.115
20	1-Phenyl-1-propanone	93-55-0	Nd	0.238
21	2-oxo-2-phenylacetaldehyde;hydrate	78146-52-8	Nd	0.130
22	2-phenylbut-2-enal	4411-89-6	Nd	0.488
23	5-methyl-2-phenylhex-2-enal	21834-92-4	Nd	0.113
24	2-phenylprop-2-enal	4432-63-7	Nd	0.034
	Total		5.125	27.368

	**Others**			
1	2,4,5-Trimethyl-1,3-dioxolane	3299-32-9	Nd	1.522
2	Dihydroxydimethylsilane	1066-42-8	Nd	0.139
3	2-Heptyl-1,3-dioxepane	4469-24-3	Nd	1.132
4	2,2,4,4,6,6-hexamethyl-1,3,5,2,4,6-trioxatrisilinane	541-05-9	0.067	Nd
5	(3E)-3-ethyl-2-methylhexa-1,3-diene	61142-36-7	0.137	Nd
6	azulene	275-51-4	0.095	Nd
7	azanium acetate	631-61-8	Nd	5.295
8	2,3-Dithiabutane	624-92-0	Nd	0.112
9	8-methoxy-2,3-dihydropyridazino[4,5-b]indol-4-one	41563-29-5	Nd	3.358
10	2-(Methoxymethyl)-2,4,5-trimethyl-1,3-dioxolane	-	Nd	0.227
11	2,3,5,6-tetramethylpyrazine	1124-11-4	Nd	0.430
12	N-methoxy-N-methylbenzamide	6919-61-5	Nd	0.262
	Total		0.300	12.478
